# La familia empoderada: Nuevas narrativas para repensar la prevención y control del dengue en Córdoba, Colombia

**DOI:** 10.18294/sc.2024.4800

**Published:** 2024-06-04

**Authors:** Nydia Nina Valencia Jiménez, Alba Zambrano Constanzo

**Affiliations:** 1 Candidata a Doctora en Estudios de Familia, Universidad de Caldas. Docente, Programa de Enfermería, Universidad de Córdoba, Montería, Colombia. nnvalencia@correo.unicordoba.edu.co Universidad de Córdoba Universidad de Córdoba Montería Colombia nnvalencia@correo.unicordoba.edu.co; 2 Médico especialista en Medicina General y Familiar. Docente, Facultad de Ciencias Médicas, Universidad Nacional de La Plata. Prosecretario de Salud de la Presidencia, Universidad Nacional de La Plata. Maestrando, Maestría en Epidemiología, Gestión y Políticas de Salud, Instituto de Salud Colectiva, Universidad Nacional de Lanús, Buenos Aires, Argentina. alba.zambrano@ufrontera.cl Universidad Nacional de Lanús Instituto de Salud Colectiva Universidad Nacional de Lanús Buenos Aires Argentina alba.zambrano@ufrontera.cl

**Keywords:** Familia, Empoderamiento, Dengue, Prevención de Enfermedades, Colombia

## Abstract

El dengue es una enfermedad que constituye un problema de salud pública difícil de controlar por la multidimensionalidad de factores asociados y las particularidades de los territorios. En este artículo se analiza la noción de empoderamiento familiar relacionada con la prevención y control del dengue en Córdoba, Colombia. De julio a octubre de 2023, a partir de un enfoque cualitativo se realizaron entrevistas semiestructuradas a 30 grupos familiares localizados en los municipios de San Andrés de Sotavento, San Bernardo del Viento, Pueblo Nuevo y Montería, seleccionados por ser territorios indígenas, afrodescendientes o con población rural y urbano en condiciones de vulnerabilidad. Los resultados muestran que las familias, más allá de sus arreglos particulares en términos de estructura y dinámica, son conscientes de su lugar en la prevención y control de enfermedades, identificando las capacidades requeridas para enfrentar el dengue. Sin embargo, reproducen narrativas de dominio conceptual y de poder que asignan un mayor compromiso por parte de los agentes educativos y de salud para el fomento de alternativas que contribuyan a disminuir los riesgos por dengue. Se discuten los desafíos que se deben enfrentar para un efectivo empoderamiento familiar, de modo que las prácticas de prevención cobren mayor vigor.

## INTRODUCCIÓN

El dengue es una de las enfermedades tropicales desatendidas que afecta mayoritariamente la salud de los grupos humanos en condición de desventaja social. La Organización Mundial de la Salud (OMS), convoca esfuerzos para construir capacidades que coadyuven a la implementación de intervenciones más coordinadas, costoeficientes y con mayores oportunidades para el aumento de cobertura, dado que, a pesar de mostrar avances en los últimos años, gran parte de las metas estipuladas en los Objetivos de Desarrollo Sostenible no se han alcanzado, por lo que es necesario continuar trabajando de cara al 2030 con estrategias integradas y mecanismos intersectoriales para su prevención y control^1^.

La OMS^2^ afirma que cerca de la mitad de la población mundial está en riesgo de enfermar; especialmente, aquellas que habitan en las regiones tropicales y subtropicales en las zonas urbanas y semiurbanas. Es necesario precisar que, aunque esta enfermedad no discrimina por género, edad o condición social, afecta especialmente a las personas, familias y comunidades con mayores niveles de vulnerabilidad social, con poco acceso a los recursos materiales, económicos, sociales y políticos que limitan sus posibilidades de desarrollo^3^^,^^4^.

Las cifras reportadas en la región de las Américas muestran evidencia acerca de la dificultad que tienen los países para combatir esta enfermedad. Para el año 2023, se reportaron un total de 4.572.765 personas infectadas con el virus del dengue (DENV). Colombia, por su parte, registró un total de 131.784 casos, ocupando el tercer lugar en la subregión Andina, antecedida por Perú (274.227) y Bolivia (156.774). Sin embargo, aportó el mayor número de casos de dengue grave (1.714) y 90 muertes confirmadas^5^. 

El Departamento de Córdoba, en Colombia, integra uno de los siete departamentos de la región Caribe, y las familias que allí habitan se encuentran en situación de alerta por dengue de forma permanente. Esto es debido a que Córdoba, junto con las cifras de otros 11 territorios, concentran el 71,4% de las notificaciones en el país. La pobreza multidimensional allí alcanza es del 36,7%, y se expresa en altos niveles de trabajo informal, escolaridad baja y rezago escolar. Adicionalmente, se presentan problemas en la prestación de los servicios de acueducto (74,6%), alcantarillado (37,2%) y recolección de basuras (55,5%). Todas estas son condiciones que deben ser consideradas en la implementación de estrategias para prevenir el dengue dado que, junto a la diversidad étnica (23,46%), suponen importantes retos para el Gobierno en materia de atención integral de la salud acorde a las particularidades de las personas, familias y comunidades^6^, evidenciándose la necesidad de profundizar en otras líneas de análisis que enriquezcan la discusión sobre el abordaje y atención integral de la enfermedad.

Si bien existen estudios que analizan el dengue desde la perspectiva clínica, epidemiológica, climática, geográfica o en la línea de los determinantes sociales de la salud, son escasos los que consideran el empoderamiento familiar, siendo más común el interés acerca del empoderamiento individual^7^^,^^8^ o comunitario^9^^,^^10^^,^^11^^,^^12^. En estos últimos estudios, se conectan los dominios de participación, liderazgo, gestión, comunicación y toma de decisiones informadas como garantes para la implementación eficiente de los programas de control de la enfermedad. 

Como se puede observar, el empoderamiento de la familia es poco explorado, convirtiéndose en un vacío de conocimientos que debilita la implementación de estrategias dirigidas a la prevención y control del dengue; más aún, cuando se cuenta con la evidencia de que a mayor acceso de recursos públicos, humanos e intelectuales mayores son las ventajas de las familias para enfrentar la enfermedad^4^; o que las deficiencias en los ejercicios democráticos, participativos y organizativos de los programas de salud se convierten en amenazas latentes del empoderamiento familiar para la prevención y control del dengue^13^. 

### Empoderamiento y salud

El empoderamiento es un concepto complejo que ha sido estudiado desde diversas disciplinas, y que ha cobrado particular notoriedad en las últimas décadas. En la actualidad, presenta diversos usos, que no siempre respetan la visión crítica de las relaciones de poder, promovida por los precursores del concepto. Podríamos identificar al menos dos aproximaciones al concepto, que presentan visiones diferentes respecto de las estructuras sociales y el rol de las personas^14^^,^^15^^,^^16^. 

Una primera aproximación es la derivada del modelo radical de empoderamiento, que se alinea con el pensamiento de Paulo Freire^17^, con el movimiento feminista radical de la década de 1970 y con todos los movimientos de corte comunitario que explotan en ese período^18^. 

Desde esta primera vertiente, se apuesta por la toma de conciencia que invita a revisar críticamente las estructuras y el sistema establecido que sostiene relaciones de dominación. El empoderamiento para los grupos de personas con mayores desventajas, en situación de exclusión o subordinación, supone un incremento de poder, el acceso al uso y control de los recursos materiales y simbólicos, y la participación en el cambio social. El empoderamiento tiene, por tanto, un carácter multidimensional que implica la superación de desigualdades o déficits a partir de un proceso de transformación y de superación de la opresión en diferentes niveles^19^.

El segundo enfoque del concepto de empoderamiento, sin cuestionar las estructuras sociales existentes, promueve que las personas y grupos en mayor desventaja incrementen la capacidad individual para ser más autónomos y autosuficientes, para depender menos de la provisión estatal de servicios o de empleo. Esto equivaldría a que estas personas adquieran mayor poder del poco que tienen. De este modo se despolitiza el concepto y el empoderamiento consistiría en que las personas sean capaces de aprovechar al máximo las oportunidades que se les presentan sin o a pesar de las limitaciones estructurales, ignorando el peso del contexto sociopolítico y del poder social^20^.

El concepto de empoderamiento tiene hoy una presencia extendida en diversos campos, entre los que se cuenta el de la salud. Si bien, la evidencia muestra la importancia de este en la promoción de la salud, prevención de las enfermedades, alfabetización sanitaria y el autocuidado^21^^,^^22^, persiste la presencia de una lógica estatal sustentada en metanarrativas sobre relaciones clientelares, mercantilista y benefactoras que actúan como ejes de la planificación y gestión sanitaria de arriba hacia abajo con énfasis en la oferta y en detrimento de la demanda ciudadana. Específicamente, se desdibuja el enfoque de empoderamiento que traduce el esfuerzo de colaborar con las personas para tomar control sobre su propia salud. 

En el campo de la salud, se ha destacado la necesidad de involucrar a las familias en los procesos de empoderamiento, dado el relevante rol que ellas cumplen en nuestra sociedad. Esta aparece como un actor clave en las tareas de autocuidado y prevención en salud. Entenderemos el empoderamiento familiar como un proceso mediante el cual las familias adquieren diversas habilidades y conocimientos, y desarrollan la confianza para tomar decisiones informadas sobre su propia vida, ejerciendo un mayor control sobre su entorno para conducir su vida en la dirección que les parezca apropiada. El empoderamiento familiar se ha asociado con una serie de beneficios para la salud y el bienestar de las familias, de allí la importancia de fortalecerlas para que puedan tomar decisiones y asuman prácticas efectivas en salud para su propio beneficio. En este caso, el empoderamiento implica ayudar a las familias a desarrollar sus habilidades, conocimientos y recursos para que puedan resolver sus propios problemas y alcanzar sus metas. Esto, sin embargo, no puede omitir el trabajo con otros niveles y actores que tienen responsabilidades de garantizar las condiciones para una vida digna de las familias^21^.

De manera particular en Colombia, la ambiciosa tarea de promover las capacidades para el empoderamiento familiar en la prevención y control de las enfermedades resulta ser cada día más compleja. Se entrecruzan los discursos de la política pública de salud en los que, por un lado, se ejerce presión para empoderar a las personas, familias, comunidades y organizaciones en temas concernientes a la participación activa, toma de decisiones y organización comunitaria de cara al fomento de la salud y, por el otro, se persiste en la imposición de barreras que limitan las posibilidades reales para que los grupos familiares accedan a las condiciones que les permitan mejorar sus realidades sanitarias^22^^,^^23^.

Asimismo, la titánica tarea de abordar enfermedades complejas, que involucran en su génesis y prevalencia la acción de vectores, requiere de la revisión minuciosa de todos los elementos en juego, que involucran aspectos culturales, económicos, relacionales y físicos. Estas enfermedades hacen parte de la historia de la humanidad desde hace aproximadamente 46 millones de años, tal como ha mostrado la evidencia^24^. El virus del dengue, en particular, es una enfermedad que constituye un problema sanitario de antigua data^25^^,^^26^^,^^27^, manteniendo su poder más allá de los históricos esfuerzos estatales para su control y vigilancia^28^.

Un aspecto relevante en las prácticas sociales, y en las prácticas en salud en particular, son las narrativas que circulan tanto en las propias familias como en las entidades responsables de brindar servicios en esta materia. Son relatos que las familias realizan e integran a sus conocimientos, creencias, experiencias e historias. Las narrativas familiares pueden tener un impacto significativo en la salud de las familias, ser el cimiento que mantiene el *statu quo*, así como también pueden sentar las bases para el cambio en dirección positiva. La exploración de las narrativas sobre empoderamiento en el campo de la salud puede aportar información para identificar las lógicas en juego, y las eventuales rutas para su transformación si ello fuese necesario. Acceder a las narrativas familiares permite la recuperación de experiencias comunicacionales, como un primer eslabón para el empoderamiento y la promoción de la salud.

Considerando lo antes expuesto, nos interesa en este artículo analizar las nociones de empoderamiento familiar relacionadas con la prevención y control del dengue en Córdoba, Colombia. 

## METODOLOGÍA

La metodología empleada en el desarrollo de esta investigación corresponde a un estudio cualitativo con enfoque narrativo. Se entiende aquí que la narrativa es una de las rutas idóneas para la producción y reproducción de conocimientos sobre el tejido relacional inherente a las dinámicas familiares que, a su vez, son un libreto que integran la vida en familia, las experiencias y la autorreferencia sobre cómo enfrentar y resistir el dengue, que se urden entre los discursos hegemónicos y aquellos construidos en la cotidianidad.

### Recolección de información y selección de las personas participantes

El trabajo de campo se implementó durante los meses de julio a octubre de 2023 en los municipios de San Bernardo del Viento, San Andrés de Sotavento, Pueblo Nuevo y Montería del Departamento de Córdoba, Colombia. Estas localidades comprenden realidades socioculturales diversas, desde donde configuran su tejido relacional las familias afrodescendientes, indígenas, campesinas y urbanas, en situación de vulnerabilidad social. Las familias se seleccionaron con un muestreo no probabilístico por conveniencia atendiendo a los siguientes criterios: grupos familiares localizados en sectores con un mayor número de casos de dengue notificados ante las respectivas secretarías de salud, con residencia permanente y autorreferencia a grupos indígenas, afrodescendientes, campesinos o familias vulnerables. 

El número de familias participantes se estableció en 30 familias, acorde al nivel de saturación teórica de las categorías^29^: ocho grupos familiares indígenas, ocho afrodescendientes, ocho campesinas y seis urbanas en condiciones de vulnerabilidad social, con un total de 170 personas entre adultos mayores, adultos, jóvenes, adolescentes y niños y niñas. Se empleó la entrevista semiestructurada para ofrecer una mayor oportunidad a los participantes de narrar, desde su experiencia, las formas relacionales para empoderarse en torno a prevenir y controlar el dengue. El instrumento se sometió a un panel de expertos y se puso a prueba con una familia para corroborar si las preguntas se comprendían. 

Se encontraron un total de 23 familias extensas conformadas por dos o tres generaciones y siete familias nucleares que respondieron a la conformación de madre, padre, hijos e hijas. La edad mínima de las personas entrevistadas fue de 8 años y la máxima de 90. En lo que respecta a los ingresos, la mayoría de las familias devenga menos de un salario mínimo legal vigente (cerca de 335 dólares mensuales).

La jefatura de la familia en su mayoría fue masculina (19 personas), cuyos grados de escolaridad más representativos fueron primaria incompleta (9), sin escolaridad (8) y técnicos (5); y, entre las principales ocupaciones se destacaron la agricultura (7), oficios varios (4), amas de casa (4), pescador (3) y empleados (3). 

Las familias del estudio residen en los municipios de San Bernardo del Viento, San Andrés de Sotavento, Pueblo Nuevo y Montería, territorios localizados en el Departamento de Córdoba de la costa Caribe de Colombia. Estos fueron seleccionados atendiendo los criterios de inclusión concernientes a la mayoría de los casos notificados de dengue, población étnica mayoritaria y grupos familiares rurales.

El trabajo de campo se realizó en varios momentos: un primer acercamiento con las autoridades sanitarias permitió obtener el registro de los casos de dengue y su distribución geográfica en el municipio, seguidamente se contactó a los líderes sociales, indígenas, afrodescendientes o campesinos para contar con el aval de acceso a las familias y la identificación de los grupos familiares. Posteriormente, se hicieron visitas domiciliaras para socializar el objetivo del proyecto y conocer la disponibilidad de la familia para participar del mismo, permitiendo la concertación para la realización de las entrevistas con presencia de todos los miembros de la familia. 

### Análisis de información

Las entrevistas tuvieron una duración aproximada de 60 minutos, fueron grabadas previa autorización y firma del consentimiento informado del representante elegido por los miembros de la familia. Cada una de las entrevistas se transcribieron como copia fiel del audio, se les aplicó las normas Jefferson^30^ y se organizaron, codificaron y categorizaron con apoyo del software ATLAS.ti (versión 23). El proceso analítico inició con la lectura de las conversaciones familiares, la segmentación de las citas libres, la agrupación por ejes temáticos que facilitaran la separación de los fragmentos correspondientes, ajustándose a los parámetros definidos por Bardin^31^, quien orienta sobre la definición de tres momentos para el análisis de contenido. 

La fase del preanálisis inicia con la lectura en profundidad del material obtenido en el trabajo de campo, la cual facilita la selección de aquellas narrativas ajustadas al objetivo del estudio. A partir de lo anterior, se da paso a la construcción del primer borrador documental bajo los criterios de exhaustividad, representatividad, homogeneidad y pertinencia. El segundo momento es la fase exploratoria, donde se procede a organizar las narrativas aplicando el ejercicio de codificación y el sistema categorial, mediante el cual se logra la transformación de los datos primarios en una serie de unidades de registro y contexto. El último momento de la fase analítica se procedió a categorizar e interpretar siguiendo fielmente las recomendaciones de Bardin^31^ sobre los procedimientos de categorización puro *a priori*, tomando en consideración las categorías que fueron tomadas de las bases teóricas del empoderamiento, así como aquellas que surgieron en el marco de las conversaciones de la familia. Cabe señalar que en el estudio emergen varios ejes de análisis, pero en este artículo nos focalizamos en uno de ellos: el significado del empoderamiento familiar (“el empoderamiento familiar: no se ve, pero se siente”). De este derivan tres subcategorías emergentes: a) un poder sobre los otros, b) es la chispa, y, c) alerta todo el tiempo.

### Aspectos éticos

Este estudio forma parte de la tesis doctoral “Empoderamiento familiar para la gestión de estrategias territoriales en la prevención y control del dengue en el Departamento de Córdoba, Colombia”, la cual fue sometida al Comité de Ética de la Universidad de Caldas, Colombia y aprobada mediante el referéndum del mes de noviembre de 2022. También, se adoptaron los lineamientos que regulan el ejercicio investigativo en Colombia, contemplados en la Resolución 8430 de 1993, para los estudios considerados sin riesgo. En todo momento se garantizó la libre participación de todos los miembros de la familia, el respeto por las ideas y por las diferencias. 

### Sesgos en el estudio y mecanismos para su control

A cada una de las etapas del estudio se le aplicó una revisión minuciosa de la metodología empleada y las interpretaciones del equipo investigador, a partir de formularnos algunos interrogantes, cuyas respuestas permitieron comprender los distintos sesgos que pudieron presentarse en el contexto de la investigación y cómo fueron controlados.

*Sesgo de selección*: para la selección de las familias se contó con el apoyo de las organizaciones sociales del territorio y algunos líderes de las minorías étnicas, quienes de forma separada hicieron un listado con las familias potenciales para el estudio. Además, se estipularon los siguientes criterios de inclusión: mayor y/o menor apropiación de saberes, prácticas, participación, gestión y comunicación en torno al manejo del dengue con miras a obtener diversas miradas del fenómeno. Todo lo anterior, permitió emplear diversos métodos de selección para mitigar el impacto en los posibles sesgos de la muestra.

*Sesgo del observador y de análisis*: el estudio contó con dos académicos externos que sirvieron como pares evaluadores de la calidad de los resultados. Cada uno leyó el contenido de las entrevistas, realizó el ejercicio de codificación y categorización, para luego hacer una triangulación de investigadores. Así, se logró reducir la influencia involuntaria que pudiese generar el equipo investigador sobre los resultados obtenidos y su análisis.

*Sesgo cultural*: el estudio contempló la inclusión de familias indígenas, afrodescendientes, campesinas y urbano vulnerables. Ello implicó el reconocimiento de las diferencias culturales desde el momento del diseño de la investigación y la inclusión de miembros con antecedentes culturales en el equipo investigativo, que facilitaron el acceso privilegiado a cada uno de los territorios seleccionados. 

## RESULTADOS

Los resultados del análisis nos permiten distinguir tres tipos de narrativas en torno al empoderamiento familiar y la prevención del dengue. La primera, muestra una visión conflictiva del poder asociada al concepto del empoderamiento como expresión de la desigualdad, en la lógica del poder sobre. La segunda narrativa, reconoce el empoderamiento como una fuerza intrínseca que se asocia a la capacidad para enfrentar problemas. La tercera narrativa, se asocia a la capacidad de enfrentar el dengue.

Los resultados del estudio se proyectan a partir de la categoría “*el empoderamiento familiar: no se ve, pero se siente”,* como una forma natural de las familias para referirse a un concepto novedoso, difícil de explicar, invisible para los otros, pero que al momento de conceptualizarlo lo sienten como parte de un proceso inherente de su cotidianidad.

Gran parte de los grupos familiares presentan una narrativa del empoderamiento que lo enlazan con las formas tradicionales de poder, dinero o mando. Estas narrativas las podemos denominar “*Un poder sobre los otros”* y se construyen desde la perspectiva del empoderamiento familiar como forma de poder, mando y control de unos sobre otros en asuntos que conciernen la vida en familia, destacándose que la figura de autoridad (padre o adultos mayores) se visibilizan como aquellas personas con autoridad para ocupar lugares de autoridad y tomar las decisiones necesarias para alcanzar el bienestar y el equilibrio, dado que tienen un mayor número de experiencias que le permiten resolver situaciones de salud, como puede leerse en los siguientes relatos:

*Bueno, yo pienso que aunque se hable de empoderamiento de la familia, siempre hay una persona que es la que tiene todo el poder de guiar al grupo familiar, es la que sabe más de la vida y ha tenido un mayor número de experiencias.* (Abuela, familia 25)

*Bueno, más o menos te diría, una familia cuando tiene una persona empoderada como es la que manda, ¿no? Es como la de capataz, la ama de la casa. Bueno, esa es una señora empoderada [risas], que es la que manda, la que tiene el control de la casa. Es la señora de la casa, porque no todos en la familia pueden mandar o decidir qué se va a hacer cuando alguien está enfermo, es una cabeza visible la que puede decir las cosas y que los demás sientan el respeto para seguirla.* (Abuela, familia 26)

*La familia empoderada es como que tenemos un poder sobre los otros, o sea, es cuando mi papá nos manda a hacer algo, ahí él tiene el poder, porque ajá los hijos tenemos que obedecer a los padres, no podemos estar contradiciendo a quien tiene el mando de la casa* [risas]. (Hijo adulto, familia 7)

Asimismo, se construyeron apreciaciones en torno al empoderamiento familiar como ejercicio del poder político, económico y social, siempre en una perspectiva de “arriba hacia abajo”, en las que un grupo social específico ejerce el dominio sobre la familia, constituyéndose en una relación consensuada y sumisa so pena de ser excluida de beneficios para su supervivencia. La familia percibe que tener vínculos con el poder les permite construir oportunidades para las nuevas generaciones, por lo que ven la necesidad de adherirse a estas formas naturales de poder entre sus comunidades para tener acceso a algunos de los recursos necesarios para su desarrollo, sus relatos así lo describen:

*Mire cuando yo pienso en empoderamiento se me viene a la cabeza el poder, puede ser el poder político o el poder de los más ricos sobre mi familia. Sin ellos, no hay nada, me entiendes… los de arriba son los que mandan y uno tiene que quedarse callado para que le dan chamba, porque de lo contrario ¿cómo mantiene uno a su familia?* (Padre, familia 15)

*Yo creo que la familia empoderada es dependiendo si tienen plata o no. Uno dice, esa gente es poderosa, bueno porque tiene plata, pero de buena aquellos que nacen con esa plata, porque uno como familia no puede empoderarse sino tiene ese dinero para hacerlo, le queda más difícil, hay que pegarse de los que sí la tienen para sacar adelante a los hijos*. (Hijo adulto, familia 19)

La segunda narrativa, se denominó *“es la chispa”*, por referirse a la fuerza interior de las familias para enfrentar los problemas. Fue común encontrar referencias al potencial interno de las personas para contribuir con el manejo de las enfermedades, incluyendo la relación con Dios como fuente de fe para controlar aquello que supera los límites naturales de la familia. A continuación, sus relatos dan cuenta de ello:

*Que tenemos como un potencial entre nosotros, entre sí mismos, pa estar dispuestos, con personalidad, pasaría a lo que sea, a buscar, a ayudar para enfrentar esa enfermedad*. (Abuelo, familia 6)

*Hombe, el ser humano debe tener la capacidad para emprender. Sí, si el ser humano no emprende va a estar ahí, o sea, debe tener una chispa para mover. Y el empoderamiento tiene esa función. Es la chispa. El empoderamiento de la población en la capacidad de de emprender, de liderar, de hacer acción*. (Padre, familia 1)

*Es como atreverse a realizar, hacer algo, gestionar algo. Es como ser, como positivo, con uno mismo. Empoderar. Empoderarse de algo. “Yo sí puedo hacer esto. Sí voy a hacer esto. Sí puedo realizar esto”. Cuando una familia está empoderada nunca dice que no. Siempre está como con la mente positiva, hacia un objetivo, eso no se habla aquí mire vamos a hacer empoderados, no eso no se habla, pero se expresa con nuestras acciones*. (Padre, familia 29)

*La familia empoderada es positiva cuando tiene un pariente enfermo. Uno no es negativo uno es positivo, uno por lo menos se aferra a Dios porque él es grande, te pasa el poder para creer que saldremos de ese problema*. (Madre, familia 9)

Una tercera narrativa aludió a la capacidad familiar para prevenir y controlar: *“alerta todo el tiempo”.* Expresa la presencia de capacidades y habilidades familiares, entre las que destacan: el liderazgo, la comunicación, el diálogo, la distribución de funciones, la educación y las prácticas familiares preventivas, como aquellos atributos requeridos para alcanzar el empoderamiento en la prevención y control del dengue:

*Es liderar, liderar esas campañas que hagan prevención contra esa enfermedad. Hay que tener empoderamiento para eso, sabes la familia es la llamada a prevenir porque es algo de nosotros mismos, es algo que se produce en la casa, pero para eso es necesario tener el liderazgo*. (Padre, familia 1)

*Los hijos siempre tiene que estar atentos a cualquier paso que den los padres, o sea, nosotros somos el reflejo de nuestros hijos, si el padre comunica o dialogan: hijo mira está pasando esto para la cuestión del dengue, para que pongas esto en práctica, para que sea para prevenir este tipo de enfermedad, ellos se encuentran en la capacidad de comprender, eso hace una familia empoderada, pero la que no, no le interesa charlar sobre esos temas*. (Padre, familia 2)

*La familia empoderada es la que está prevenida contra el dengue. Está alerta todo el tiempo, porque cuando pasa la moda del dengue bajamos la guardia, dejamos de hablar de la enfermedad, y otra vez llega y nos ataca. Creo que la familia debe mantener un diálogo siempre del tema así estamos pilas y enseñamos a nuestros hijos a enfrentar esa enfermedad tan peligrosa*. (Nuera, familia 3)

*Pues buscar formas de solucionar el problema del dengue y siempre estar atento al tema, porque una familia que no se mantenga alerta de todos los peligros se deja coger ventajas de los problemas. Pero la forma de estar alerta es comunicando y poniendo en práctica lo que aprendemos, creo que eso es lo más difícil, pero con el empoderamiento podemos lograrlo*. (Joven, familia 30)

*Bueno, cuando una familia está empoderada las tareas se dividen, porque mientras uno hace una cosa, el otro va camino a hacer la otra, pero cuando en la familia todo se le deja a una sola persona no se logra combatir nada, creo que paso al revés, se agrandan los problemas de salud*. (Abuela, familia 19)

Un aspecto que vale la pena resaltar es la alusión de la familia al liderazgo, la información y la capacidad de persuación como alternativas para motivar a otras familias en determinados temas que maneja con experticia determinada familia, como puede observarse en el siguiente relato:

*La familia empoderada es aquella que están siempre de primero, entonces una familia líder es la siempre arranca desde la cabeza y ya tiene seguidores en eso que necesita hacer. La familia que lidera se empodera primero después sigue otra, que tiene un rango más bajo pero siempre están ahí al pendiente de aquellos que llevan la delantera*. (Joven, familia 23)

En este sentido, el empoderamiento familiar se enuncia desde la colectividad, el trabajo con los otros y la apropiación conceptual de un tema en específico. Hecho que trasciende la vivienda para instalarse en un plano social que requiere la unión de las familias para el logro de objetivos específicos. 

En síntesis, se pudo observar que las narrativas sobre empoderamiento familiar se producen con la misma intensidad en los diferentes tipos de familia entrevistados y no se observaron diferencias significativas entre los grupos familiares, siendo más potente el discurso de los padres o adultos mayores que en la población joven, adolescente o niños y niñas. Solo se encontraron excepciones en aquellas narrativas producidas por las familias campesinas o inscritas en minorías étnicas en torno a la solidaridad, la unión y el trabajo en equipo a diferencia de aquellas localizadas en contextos urbanos donde prima más la desconfianza, el desinterés por participar y la apatía para gestionar programas de prevención y control. 

## DISCUSIÓN

Gran parte de los relatos familiares se refieren al empoderamiento en la perspectiva del poder desde la dimensión del dinero, la toma de decisiones y la voz de mando a nivel intrafamiliar y en el entorno inmediato. De igual manera, surgió el enfoque subjetivo del empoderamiento cuando se pudo evidenciar la alusión al potencial interno y las habilidades para actuar; y, en una tercera línea discursiva se hizo visible la noción de capacidades familiares en términos de liderazgo, comunicación, distribución de funciones, toma de decisiones, educación familiar y las prácticas preventivas de la familia en torno al dengue. 

Las familias reiteran la noción de poder que se viene debatiendo hace algunas décadas en torno al ejercicio de dominación de un grupo sobre otro, o la influencia ejercida por una persona para obligar a otra a que realice acciones en contra de su voluntad denominada “poder sobre”. Asimismo, los grupos familiares aludieron a otra de las variantes de esta perspectiva, cuando se refieren a la elevación del poder sin la mediación del conflicto o daño aparente a otra persona, conocida como “poder para”^32^. Este tipo de narrativas que construyen las familias en torno al empoderamiento hacen parte de lo que Lidenmann^33^ denomina *“la historia socialmente compartida”* o “*narrativas maestras”*, determinando en gran medida aquellos comportamientos aceptados en la sociedad, que se siguen reproduciendo a nivel familiar aun cuando actúan como dispositivos para la amplitud de las disparidades sanitarias y de injusticia social^34^. 

El poder al interior de la familia se hizo visible con la referencia a la autoridad y el mando de los hombres como proveedores económicos. La manera de comprender estos hallazgos es leerlos desde el contexto sociocultural de las familias entrevistadas, caracterizado por situaciones de pobreza extrema, bajos niveles educativos, trabajos informales, familias extensas y la jefatura masculina^3^^,^^4^. De este modo, el empoderamiento familiar se ve amenazado porque de acuerdo con los resultados de Ramírez *et al*.^35^, el empoderamiento está definido por las posibilidades de adquirir recursos del contexto para la toma de decisiones libres e informadas, así como la capacidad de negociación y una mayor libertad de movilización y asociación. 

Esta postura entra en diálogo con los planteamientos de Serrano^36^ en términos del cuestionamiento sobre la posibilidad de un empoderamiento genuino cuando media un tipo de sociedad conservadora, a partir de la cual, las familias entrevistadas contarían con limitaciones para desarrollar este tipo de procesos, en la medida en que se impone la figura individualista dominante de un padre, o como bien lo expresaron en la idea de un “capataz”, cuya analogía es común en la región ganadera y agrícola donde se desarrolló el estudio. No obstante, de forma paralela, las familias entrevistadas construyen narrativas en las que, si bien la mujer acepta la autoridad masculina, paso a paso va desplegando sus capacidades para la prevención y control de enfermedades que les permite tomar decisiones y movilizar sus saberes. De forma similar a los resultados de Arias *et al*.^37^ y Madero *et al*.^38^, esta interfaz representa un lugar de la mujer, en el que a pesar de la carga social conferida, a partir del ejercicio de sus funciones, ellas logran mayores niveles de empoderamiento. 

Las familias también se refirieron al poder político y económico circulante en sus entornos como dispositivos de control por encima del empoderamiento familiar. La coincidencia en sus narrativas en torno a la necesidad del dinero para lograr alcanzar sus metas y propósitos, al igual que aferrarse a la imagen política como medio para satisfacer necesidades de empleo o de supervivencia, es fiel reflejo de las características sociopolíticas del territorio, donde prima el control sobre los recursos por parte de un grupo, la fragilidad organizacional y la invisibilización de actores sociales como la familia y la comunidad^10^. Todo ello actúa en detrimento de las capacidades para el aprovechamiento de las oportunidades presentes en el territorio, porque según Zambrano y Henríquez^16^, estas condiciones fortalecen el individualismo y ven crecer relaciones clientelares con los gobiernos locales. 

Un segundo eje analítico, lo constituye la visión individualista del empoderamiento sustentada en aquellas narrativas familiares que dieron origen al rótulo categorial: “*es la chispa*”, para referirse al potencial que poseen las familias para emprender acciones, la confianza en sí mismas e incluso la fe en Dios para sortear las enfermedades. Esta perspectiva surgió en las décadas del 1970 y 1980 del siglo XX, como parte inherente del discurso del bienestar humano que motivó la lectura del vínculo con la autoestima o la realización personal en respuesta de las necesidades humanas para interactuar o relacionarse entre sí^39^.

Al respecto, Cooke^40^ plantea la lectura de la visión individualista del empoderamiento como un concepto que ha recorrido distintas formas interpretativas que van desde la autoexpresión, automejora, hasta llegar al campo de la psicología en forma de autodeterminación de la vida de sí mismo y sensación de control personal. Este proceso no se queda encapsulado en el ámbito personal, como puede leerse en los relatos de las familias entrevistadas cuando expresan la conexión de ese poder personal con la necesidad de gestionar asuntos de interés para la familia, que desde la posición de Rappaport^14^ se traduciría en contar con el poder para hacer algo mediado por una condición de dominio en función a ese aspecto en particular. 

A partir de la lectura realizada por Rappaport^14^, la noción de empoderamiento estaría integrada por dos elementos esenciales: la determinación individual y la participación democrática. El primer elemento responde al control que ejerce cada persona sobre su propia vida, y el segundo integra la participación democrática que se produce al interior de una comunidad, la cual buscan, por lo general, la defensa de los derechos legales, poder político entre otros, a partir de la interconexión de los participantes con las organizaciones como, por ejemplo, la escuela, iglesia y diferentes grupos sociales.

La lectura de Sánchez^39^ enriquece el diálogo de las narrativas familiares en Córdoba al establecer la existencia de dos formas de empoderamiento psicológico: subjetiva y potencial. La primera constituye un aspecto de interés para el logro de la competencia efectiva, por tratarse de una condición inicial que permite a las personas desarrollar actuaciones colectivas para el logro de los objetivos. Esta dimensión se potencia, en sintonía con los aportes de Zimmerman^15^ al concepto de empoderamiento, en la medida en que las personas sienten la necesidad de liberarse de los efectos de la opresión, bajo la premisa de que la individualidad requiere desarrollar el sentido del “yo” para elevar la capacidad individual y de autoconfianza.

Hasta este punto, las narrativas familiares construidas en torno al empoderamiento por parte de las familias entrevistadas se muestran concordantes con la interpretación de Ryynänen y Nivala^41^, quienes señalan dos corrientes de pensamiento para el análisis de esta categoría: la primera de corte estructuralista relacionada con el concepto de poder, y la segunda que la ubica en el marco de la fuerza interior e individual de las personas permitiéndoles actuar sobre una situación en particular. 

Por otra parte, las narrativas se enfocaron en “el ejemplo” como mediación educativa para alcanzar niveles óptimos de prevención y control del dengue. Esto es, a partir de instaurar canales de diálogo, despertar la atención y fomentar las prácticas preventivas a partir del modelaje que realizan los padres y adultos mayores. Estos aspectos resumen la intencionalidad de actuar frente a un problema presente en el territorio, que expone la toma de conciencia, la distribución de tareas y el liderazgo familiar intra y con el entorno inmediato acompañados de la habilidad de persuasión, mediante la cual una familia motivada y con apropiación conceptual sobre la temática estaría en capacidad de jalonar procesos de empoderamiento, no solo de su grupo, sino de otras familias de su comunidad. Estos hallazgos son similares a los de Garrido *et al*.^42^, en torno a la necesidad que tienen los grupos familiares de adquirir conocimientos para lograr mayores niveles de empoderamiento. 

Tal como reflexiona Sánchez Vidal^39^, la incorporación explícita del componente del poder, introducido por el concepto de empoderamiento, ha estado centrada básicamente en la dimensión del empoderamiento subjetivo de los individuos en espacios microsociales, postergando las influencias del contexto sociopolítico más amplio. Si bien esto puede ser un aporte, es necesario ser crítico respecto de los alcances de los cambios que permite una sociedad globalizada y dualizada. Por tanto, en el campo de la prevención, debemos abordar el empoderamiento en esta doble dimensión. Nos parece particularmente relevante, por ejemplo, dinamizar procesos que permitan cuestionar y transformar los roles de género, así como la necesidad de la organización y participación colectiva para avanzar en transformaciones más profundas que sostengan un sistema de prevención basado en cambios sustantivos de las condiciones de vida.

## CONCLUSIONES

En términos generales, las narrativas de empoderamiento construida por los grupos familiares permanecen ancladas a una metanarrativa perpetuada por las relaciones de dominio/sumisión que coarta la toma de decisiones, la movilización de saberes y las prácticas familiares de prevención y control de enfermedades. Estas relaciones se perciben a nivel micro cuando a pesar del lugar protagónico de la mujer en el cuidado de la salud familiar, asume con resignación la jefatura masculina por los nexos con la provisión económica; y a nivel comunitario, se observa adherencia al poder político u organizacional derivado de la necesidad de obtener beneficios económicos. 

Pese a lo anterior, en materia de prevención y control del dengue se destacan las narrativas orientadas a las capacidades del grupo familiar para enfrentar el dengue. Ello muestra claramente los dominios para que se desarrolle un proceso de empoderamiento a nivel de la familia como son el liderazgo, la comunicación intrafamiliar, la comprensión del fenómeno dengue, mantenerse alerta de forma permanente, dialogar y movilizar los saberes construidos en la colectividad a su mundo cotidiano. Estos resultados controvierten las metanarrativas del sector salud que vienen perpetuando la creencia de la imagen familiar asociada con la pasividad, sumisión y desinterés, sirviendo como punto de partida para incitar la reflexión sobre nuevas formas de abordar y atender la enfermedad. 

El desafío es construir también los mecanismos de participación, comunicación y gobernanza familiar para ofrecer los escenarios mediante los cuales las familias puedan desplegar sus capacidades en materia de prevención y control del dengue. Esto requiere el desplazamiento de metanarrativas que guían una política pública sanitaria enfocada en el control químico de vectores con poca efectividad en el desarrollo de procesos educativos familiares para invertir el sentido individualista de la intervención, por acciones colectivas donde la familia reciba el acompañamiento requerido en su camino para empoderarse. 

En síntesis, la principal limitación del estudio se deriva de la naturaleza misma de los estudios cualitativos, siendo conscientes que la extrapolación de nuestros hallazgos debe hacerse con cautela cuando se aborden otros grupos familiares del territorio, cuyas características sociodemográficas no encuadren con las familias que participaron en la presente investigación. Aun así, sería posible considerar, como un aporte a la comprensión del fenómeno estudiado, los valores de asociación y gobernanza presentes en los grupos familiares campesinos, además de la amplia participación de personas adultas mayores portadoras de saberes y experiencias enriquecidas en el ámbito del manejo de las enfermedades, que demandan otros estudios en el campo del empoderamiento familiar.
