# Supplementary material for: La familia empoderada: Nuevas narrativas para repensar la prevención y control del dengue en Córdoba, Colombia
Source: Salud Colect. 2024 Jun 4;20:e4800. [Article in Spanish] doi: 10.18294/sc.2024.4800 (PMC11925735; doi:10.18294/sc.2024.4800)
Supplement: Translation

# Translation: The empowered family: New narratives to rethink dengue prevention and control in Córdoba, Colombia

**Authors:** Nydia Nina Valencia Jiménez, Alba Zambrano Constanzo

**Affiliations:** 1 PhD Candidate in Family Studies, Universidad de Caldas. Professor, Nursing Program, Universidad de Córdoba, Montería, Colombia. nnvalencia@correo.unicordoba.edu.co; 2 PhD in Social Psychology. Academic, Department of Psychology, Universidad de La Frontera, Temuco, Chile.

**Keywords:** Family, Empowerment, Dengue, Disease Prevention, Colombia

## Abstract

Dengue is a disease that constitutes a public health problem difficult to control due to the multidimensionality of associated factors and the particularities of the territories. This article analyzes the notion of family empowerment related to the prevention and control of dengue in Córdoba, Colombia. From July to October 2023, using a qualitative approach, semi-structured interviews were conducted with 30 family groups located in the municipalities of San Andrés de Sotavento, San Bernardo del Viento, Pueblo Nuevo, and Montería, selected for being indigenous, Afro-descendant, or rural and urban populations in vulnerable conditions. The results show that families, beyond their particular arrangements in terms of structure and dynamics, are aware of their role in the prevention and control of diseases, identifying the capacities required to face dengue. However, they reproduce narratives of conceptual and power dominance that assign a greater commitment on the part of educational and health agents to promote alternatives that contribute to reducing the risks of dengue. The challenges that must be faced for effective family empowerment are discussed, so that prevention practices gain greater strength.

## INTRODUCTION

Dengue is one of the neglected tropical diseases that mainly affects the health of socially disadvantaged groups. The World Health Organization (WHO) calls for efforts to build capacities that contribute to the implementation of more coordinated, cost-effective interventions with greater opportunities for increasing coverage. Despite showing progress in recent years, many of the targets set in the Sustainable Development Goals have not been achieved. Therefore, it is necessary to continue working towards 2030 with integrated strategies and intersectoral mechanisms for its prevention and control.^1^

The WHO^2^ states that nearly half of the world’s population is at risk of getting sick, especially those living in tropical and subtropical regions in urban and semi-urban areas. It is important to note that although this disease does not discriminate by gender, age, or social condition, it particularly affects individuals, families, and communities with higher levels of social vulnerability, with limited access to material, economic, social, and political resources that constrain their development opportunities.^3^^,^^4^

The figures reported in the Americas region provide evidence of the difficulty countries face in combating this disease. By the year 2023, a total of 4,572,765 people were reported infected with the dengue virus (DENV). Colombia, on its part, recorded a total of 131,784 cases, ranking third in the Andean subregion, preceded by Peru (274,227) and Bolivia (156,774). However, Colombia contributed the highest number of severe dengue cases (1,714) and 90 confirmed deaths.^5^

The Department of Córdoba, in Colombia, is one of the seven departments in the Caribbean region, and the families living there are in a permanent state of alert due to dengue. This is because Córdoba, along with figures from 11 other territories, accounts for 71.4% of the notifications in the country. Multidimensional poverty reaches 36.7%, expressed in high levels of informal labor, low educational attainment, and school dropout rates. Additionally, there are issues with the provision of water supply services (74.6%), sewage systems (37.2%), and waste collection (55.5%). All these conditions must be considered in the implementation of strategies to prevent dengue, as they, along with ethnic diversity (23.46%), pose significant challenges for the government in providing comprehensive healthcare tailored to the particularities of individuals, families, and communities.^6^ This underscores the need to delve into other lines of analysis that enrich the discussion on the approach and comprehensive care for the disease.

While there are studies that analyze dengue from a clinical, epidemiological, climatic, geographical perspective, or along the lines of the social determinants of health, few consider family empowerment, with more common interest in individual^7^^,^^8^ or community empowerment.^9^^,^^10^^,^^11^^,^^12^ In these latter studies, the domains of participation, leadership, management, communication, and informed decision-making are connected as guarantors for the efficient implementation of disease control programs.

As can be observed, family empowerment is little explored, creating a knowledge gap that weakens the implementation of strategies aimed at the prevention and control of dengue; even more so, when there is evidence that greater access to public, human, and intellectual resources enhances families’ advantages in facing the disease;^4^ or that deficiencies in the democratic, participatory, and organizational exercises of health programs become latent threats to family empowerment for dengue prevention and control.^13^

### Empowerment and Health

Empowerment is a complex concept that has been studied from various disciplines and has gained particular prominence in recent decades. Currently, it presents diverse uses, which do not always respect the critical view of power relations promoted by the precursors of the concept. We can identify at least two approaches to the concept, which present different views regarding social structures and the role of individuals.^14^^,^^15^^,^^16^

A first approach is derived from the radical model of empowerment, which aligns with the thinking of Paulo Freire,^17^ the radical feminist movement of the 1970s, and all the community-based movements that emerged during that period.^18^

From this first perspective, it advocates for consciousness-raising that invites a critical review of the structures and established system that sustain relations of domination. Empowerment for groups of people with greater disadvantages, in situations of exclusion or subordination, involves an increase in power, access to the use and control of material and symbolic resources, and participation in social change. Therefore, empowerment has a multidimensional character that involves overcoming inequalities or deficits through a process of transformation and overcoming oppression at different levels.^19^

The second approach to the concept of empowerment, without questioning existing social structures, promotes that individuals and groups at a greater disadvantage increase their individual capacity to be more autonomous and self-sufficient, to rely less on state provision of services or employment. This would mean that these individuals gain more power from the little they have. In this way, the concept is depoliticized, and empowerment would mean that people are able to make the most of the opportunities presented to them without, or despite, structural limitations, ignoring the weight of the sociopolitical context and social power.^20^

The concept of empowerment is now widely present in various fields, including health. Although evidence shows its importance in health promotion, disease prevention, health literacy, and self-care,^21^^,^^22^ there persists a state logic sustained by metanarratives about clientelist, mercantilist, and benefactor relationships that act as the axes of top-down health planning and management, emphasizing supply to the detriment of citizen demand. Specifically, the empowerment approach that translates to collaborating with people to take control over their own health becomes blurred.

In the field of health, the need to involve families in empowerment processes has been highlighted, given the significant role they play in our society. Families emerge as key actors in self-care and health prevention tasks. We will understand family empowerment as a process through which families acquire various skills and knowledge, and develop the confidence to make informed decisions about their own lives, exercising greater control over their environment to lead their lives in the direction they deem appropriate. Family empowerment has been associated with a range of benefits for the health and well-being of families, hence the importance of strengthening them so they can make decisions and adopt effective health practices for their own benefit. In this case, empowerment involves helping families develop their skills, knowledge, and resources so they can solve their own problems and achieve their goals. This, however, cannot overlook working with other levels and actors who have responsibilities to ensure the conditions for a dignified life for families.^21^

Particularly in Colombia, the ambitious task of promoting capacities for family empowerment in the prevention and control of diseases is becoming increasingly complex. The discourses of public health policy intersect, where, on one hand, pressure is exerted to empower individuals, families, communities, and organizations on issues concerning active participation, decision-making, and community organization towards health promotion, and on the other hand, there persists the imposition of barriers that limit the real possibilities for family groups to access conditions that allow them to improve their health realities.^21^^,^^23^

Likewise, the titanic task of addressing complex diseases, which involve vectors in their genesis and prevalence, requires a thorough review of all the elements at play, involving cultural, economic, relational, and physical aspects. These diseases have been part of human history for approximately 46 million years, as evidence has shown.^24^ The dengue virus, in particular, is a disease that constitutes a long-standing health problem,^25^^,^^26^^,^^27^ maintaining its power beyond historical state efforts for its control and surveillance.^28^

A relevant aspect in social practices, and particularly in health practices, are the narratives circulating both within families themselves and in the entities responsible for providing services in this field. These are stories that families create and integrate into their knowledge, beliefs, experiences, and histories. Family narratives can have a significant impact on the health of families, serving as the foundation that maintains the status quo, as well as laying the groundwork for positive change. Exploring narratives about empowerment in the field of health can provide information to identify the underlying logics at play, and potential pathways for transformation if necessary. Accessing family narratives allows for the recovery of communicational experiences, as a first step towards empowerment and health promotion.

Taking into account the aforementioned, this article aims to analyze the notions of family empowerment related to the prevention and control of dengue in Córdoba, Colombia.

## METHODOLOGY

The methodology employed in the development of this research corresponds to a qualitative study with a narrative approach. Here, narrative is understood as one of the suitable paths for the production and reproduction of knowledge about the relational fabric inherent in family dynamics, which, in turn, are a script that integrates family life, experiences, and self-reference on how to confront and resist dengue, woven between hegemonic discourses and those constructed in everyday life.

### Data Collection and Selection of Participants

The fieldwork was implemented during the months of July to October 2023 in the municipalities of San Bernardo del Viento, San Andrés de Sotavento, Pueblo Nuevo, and Montería in the Department of Córdoba, Colombia. These locations encompass diverse sociocultural realities, where Afro-descendant, indigenous, peasant, and urban families, in situations of social vulnerability, shape their relational fabric. Families were selected using a non-probabilistic convenience sampling method based on the following criteria: family groups located in areas with a higher number of reported dengue cases to the respective health secretariats, with permanent residence and self-identification as indigenous, Afro-descendant, peasant, or vulnerable families.

The number of participating families was established at 30 families, according to the theoretical saturation level of the categories:^29^ eight indigenous family groups, eight Afro-descendant, eight peasant, and six urban families in conditions of social vulnerability, totaling 170 individuals including elderly, adults, youth, adolescents, and children. A semi-structured interview was employed to provide participants with a greater opportunity to narrate, from their experience, the relational ways to empower themselves in preventing and controlling dengue. The instrument underwent expert panel review and was tested with one family to verify if the questions were understood.

A total of 23 extended families, consisting of two or three generations, and seven nuclear families, composed of mother, father, sons, and daughters, were identified. The minimum age of the interviewees was 8 years old, and the maximum was 90. Regarding income, the majority of families earned less than one minimum legal monthly wage (approximately 335 dollars).

The head of the family was mostly male (19 individuals), with the most representative educational levels being incomplete primary education (9), without schooling (8), and technical (5); and among the main occupations, agriculture (7), various trades (4), housewives (4), fishermen (3), and employees (3) stood out.

The families in the study reside in the municipalities of San Bernardo del Viento, San Andrés de Sotavento, Pueblo Nuevo, and Montería, territories located in the Department of Córdoba on the Caribbean coast of Colombia. These were selected based on inclusion criteria related to the majority of reported dengue cases, majority ethnic population, and rural family groups.

Fieldwork was conducted in several stages: an initial approach with health authorities facilitated the acquisition of dengue case registries and their geographical distribution within the municipality. Subsequently, social, indigenous, Afro-descendant, or peasant leaders were contacted to obtain approval for access to families and to identify family groups. Then, home visits were made to socialize the project’s objectives and assess the family’s willingness to participate, allowing for the coordination of interviews with all family members present.

### Data Analisys

The interviews lasted approximately 60 minutes each and were recorded with prior authorization and informed consent signed by the representative chosen by the family members.. Each interview was transcribed verbatim from the audio recording, following the Jeffersonian conventions,^30^, and were organized, coded, and categorized using the ATLAS.ti software (version 23). The analytical process began with the reading of the family conversations, segmenting the free quotes, grouping them by thematic axes to facilitate the separation of corresponding fragments, following the parameters defined by Bardin,^31^ who guides on the definition of three moments for content analysis.

The pre-analysis phase begins with an in-depth reading of the material obtained in the fieldwork, which facilitates the selection of narratives aligned with the study’s objectives. From this, the construction of the first draft document is initiated following criteria of exhaustiveness, representativeness, homogeneity, and relevance. The second moment is the exploratory phase, where narratives are organized through coding and categorical system exercises, transforming primary data into a series of recording and contextual units. The last moment of the analytical phase involved categorization and interpretation, faithfully following Bardin’s recommendations^31^ on pure a priori categorization procedures, considering categories taken from theoretical empowerment bases as well as those emerging from family conversations. It is worth noting that several axes of analysis emerge in the study, but this article focuses on one: the meaning of family empowerment (“family empowerment: it’s not seen, but it’s felt”). From this, three emerging subcategories derive: a) power over others, b) it’s the spark, and, c) alert all the time.

### Ethical aspects

This study is part of the doctoral thesis “Family Empowerment for the Management of Territorial Strategies in the Prevention and Control of Dengue in the Department of Córdoba, Colombia,” which was submitted to the Ethics Committee of the University of Caldas, Colombia, and approved by referendum in November 2022. Additionally, the guidelines regulating research in Colombia, as outlined in Resolution 8430 of 1993 for studies considered low-risk, were adopted. At all times, the free participation of all family members was ensured, along with respect for their ideas and differences.

### Biases in the Study and Mechanisms for Control

At each stage of the study, a thorough review of the methodology used and the interpretations of the research team was conducted by formulating some questions. The answers to these questions helped to understand the different biases that may have arisen in the context of the research and how they were controlled.

*Selection Bias:* For the selection of families, we relied on the support of social organizations in the territory and some leaders of ethnic minorities, who separately compiled lists of potential families for the study. In addition, the following inclusion criteria were established: greater and/or lesser appropriation of knowledge, practices, participation, management, and communication regarding dengue management, with the aim of obtaining diverse perspectives on the phenomenon. All of the above allowed for the use of various selection methods to mitigate the impact of possible biases in the sample.

*Observer and Analysis Bias:* The study involved two external academics who served as peer reviewers to assess the quality of the results. Each one read the content of the interviews, conducted the coding and categorization exercise, and then performed a researcher triangulation. Thus, the unintentional influence that the research team could have on the results obtained and their analysis was reduced.

*Cultural Bias:* The study included indigenous, Afro-descendant, peasant, and urban vulnerable families. This involved recognizing cultural differences from the design stage of the research and including members with cultural backgrounds in the research team, who facilitated privileged access to each of the selected territories.

## RESULTS

The results of the analysis allow us to distinguish three types of narratives regarding family empowerment and dengue prevention. The first one presents a conflicting view of power associated with the concept of empowerment as an expression of inequality, in the logic of power over. The second narrative recognizes empowerment as an intrinsic force associated with the ability to confront problems. The third narrative is associated with the ability to confront dengue.

The results of the study are projected based on the category “family empowerment: it’s not seen, but it’s felt,” as a natural way for families to refer to a novel concept, difficult to explain, invisible to others, but when conceptualizing it, they feel it as part of an inherent process in their daily lives.

Many of the family groups present a narrative of empowerment that links it to traditional forms of power, money, or command. These narratives can be termed “*Power over others*” and are constructed from the perspective of family empowerment as a form of power, command, and control over others in matters concerning family life. It is noteworthy that the authority figure (father or elderly adults) are visible as those with authority to occupy positions of authority and make necessary decisions to achieve well-being and balance, as they have a greater number of experiences that allow them to resolve health situations, as can be seen in the following narratives:

*Well, I think that even though we talk about family empowerment, there is always one person who has all the power to guide the family group, the one who knows more about life and has had a greater number of experiences.* (Grandmother, Family 25)

*Well, more or less, I would say, in a family when there is an empowered person who is the one in charge, right? It’s like the foreman, the lady of the house. Well, that’s an empowered lady [laughs], who is the one in charge, the one who has control of the house. She’s the lady of the house because not everyone in the family can give orders or decide what to do when someone is sick. It’s a visible head who can say things and the others feel the respect to follow her.* (Grandmother, Family 26)

*The empowered family is like when we have power over others, I mean, it’s when my dad tells us to do something, there he has the power, because, you know, the children have to obey the parents, we can’t be contradicting the one who’s in charge of the house* [laughs]. (Adult son, Family 7)

Likewise, assessments were made regarding family empowerment as an exercise of political, economic, and social power, always from a “top-down” perspective, in which a specific social group exercises dominance over the family, constituting a relationship of consensus and submission under threat of being excluded from benefits for their survival. Families perceive that having connections with power allows them to build opportunities for future generations, so they see the need to adhere to these natural forms of power within their communities to access some of the resources necessary for their development, as their accounts describe:

*When I think of empowerment, I associate it with power, it could be political power or the power of the richest over my family. Without them, there is nothing, you understand... those at the top are the ones who give orders and one has to stay quiet to get a job, because otherwise, how does one support their family*? (Father, Family 15)

*I believe that an empowered family depends on whether they have money or not. You say, those people are powerful, well, because they have money, but lucky are those who are born with that money, because as a family, you can’t empower yourself if you don’t have that money to do it, it becomes more difficult, you have to stick to those who do have it to support your children.* (Adult son, Family 19)

The second narrative was termed *“it’s the spark”* referring to the inner strength of families to face problems. It was common to find references to the internal potential of individuals to contribute to the management of diseases, including the relationship with God as a source of faith to control what surpasses the natural limits of the family. The following accounts reflect this:

*We have a potential among us, within ourselves, to be willing, with personality, to do whatever it takes, to search, to help to face that illness.* (Grandfather, Family 6)

*Man, humans must have the capacity to undertake. Yes, if humans don’t undertake, they’ll just be there, I mean, they must have a spark to move. And empowerment serves that purpose. It’s the spark. Empowerment of the population in the capacity to undertake, to lead, to take action.* (Father, Family 1)

*It’s like daring to do, to accomplish something, to manage something. It’s like being positive, with oneself. Empowering. Empowering oneself with something. ‘I can do this. I will do this. I can accomplish this.’ When a family is empowered, they never say no. They always have a positive mindset, towards a goal. We don’t talk here about being empowered, no, that’s not discussed, but it’s expressed through our actions.* (Father, Family 29)

*The empowered family is positive when they have a sick relative. You’re not negative, you’re positive, you at least cling to God because He is great, He gives you the power to believe that we will overcome this problem.* (Mother, Family 9)

A third narrative referred to the family’s ability to prevent and control: *“alert all the time”.* It expresses the presence of family capacities and skills, including leadership, communication, dialogue, task distribution, education, and preventive family practices, as those attributes required to achieve empowerment in dengue prevention and control:

*It’s about leading, leading those campaigns for prevention against that disease. You need empowerment for that, you know, the family is the one called to prevent because it’s something of ourselves, it’s something that happens in the house, but for that, leadership is necessary.* (Father, Family 1)

*The children always have to be attentive to any steps taken by the parents, I mean, we are the reflection of our children, if the father communicates or dialogues: ‘son, look, this is happening for the dengue issue, so that you can put this into practice, to prevent this type of disease,’ they are able to understand. That’s what makes an empowered family, but those who are not, are not interested in discussing those topics.* (Father, Family 2)

*The empowered family is the one that is prepared against dengue. They are alert all the time because when the dengue trend passes, we let our guard down, we stop talking about the disease, and then it comes back and attacks us again. I believe that the family should always maintain a dialogue on the topic so that we are alert and teach our children to face this very dangerous disease.* (Daughter-in-law, Family 3)

*Well, to look for ways to solve the dengue problem and always be attentive to the issue because a family that doesn’t stay alert to all dangers lets problems take advantage of them. But the way to stay alert is by communicating and putting into practice what we learn. I think that’s the hardest part, but with empowerment, we can achieve it.* (Young person, Family 30)

*Well, when a family is empowered, tasks are divided because while one person does one thing, the other is on their way to do another. But when everything is left to one person in the family, nothing is achieved in combating anything. I think it’s the other way around; health problems become bigger.* (Grandmother, Family 19)

One aspect worth highlighting is the family’s reference to leadership, information, and persuasive ability as alternatives to motivate other families on certain topics that a particular family handles with expertise, as can be observed in the following account:

*The empowered family is the one that is always at the forefront, so a leading family is the one that always starts from the top and already has followers in what needs to be done. The family that leads empowers itself first, then another follows, which has a lower rank but always stays attentive to those who are leading.* (Young person, Family 23)

In this sense, family empowerment is articulated through collectivity, collaboration with others, and the conceptual appropriation of a specific issue. This goes beyond the household to establish itself on a social level that necessitates the unity of families to achieve specific objectives.

In summary, it was observed that narratives about family empowerment are produced with the same intensity across different types of families interviewed, and no significant differences were observed between family groups. The discourse of parents or older adults was more powerful than that of young people, teenagers, or children. Exceptions were found in narratives produced by peasant families or those belonging to ethnic minorities, which emphasized solidarity, unity, and teamwork, unlike those in urban contexts where distrust, lack of interest in participation, and apathy towards managing prevention and control programs are more prevalent.

## DISCUSSION

Much of the family narratives refer to empowerment from the perspective of power in terms of money, decision-making, and leadership within the family and immediate environment. Similarly, a subjective approach to empowerment emerged when allusion was made to internal potential and the ability to take action. Additionally, a third discursive line made visible the notion of family capacities in terms of leadership, communication, distribution of tasks, decision-making, family education, and preventive practices regarding dengue fever.

Families reiterate the notion of power that has been debated for decades regarding the exercise of domination of one group over another, or the influence exerted by one person to compel another to act against their will, known as “power over.” Similarly, family groups alluded to another variant of this perspective when they referred to the elevation of power without the mediation of conflict or apparent harm to another person, known as “power to”.^32^ These types of narratives constructed by families regarding empowerment are part of what Lidenmann^33^ calls *“socially shared history”* or *“master narratives”*, largely determining those behaviors accepted in society that continue to be reproduced at the family level, even as they serve as mechanisms for the perpetuation of health disparities and social injustice.^34^

Power within the family became evident with reference to the authority and leadership of men as economic providers. Understanding these findings entails reading them within the sociocultural context of the interviewed families, characterized by situations of extreme poverty, low educational levels, informal employment, extended families, and male-headed households.^3^^,^^4^ Thus, family empowerment is threatened because, according to the results of Ramírez et al.,^35^ empowerment is defined by the ability to acquire resources from the context for making free and informed decisions, as well as the capacity for negotiation and greater freedom of movement and association.

This stance enters into dialogue with the proposals of Serrano^36^ in terms of questioning the possibility of genuine empowerment when a conservative society mediates. According to this perspective, the interviewed families would face limitations in developing such processes, as the dominant individualistic figure of a father is imposed, or as expressed in the idea of a “foreman,” whose analogy is common in the livestock and agricultural region where the study was conducted. However, in parallel, the interviewed families construct narratives in which, although women accept male authority, step by step they unfold their capacities for disease prevention and control, allowing them to make decisions and mobilize their knowledge. Similar to the findings of Arias et al.^37^ and Madero et al.,^38^ this interface represents a space for women where, despite the social burden conferred, through the exercise of their functions, they achieve higher levels of empowerment.

Families also referred to the political and economic power circulating in their environments as control devices overriding family empowerment. The consensus in their narratives regarding the need for money to achieve their goals and purposes, as well as clinging to political influence as a means to satisfy employment or survival needs, is a faithful reflection of the socio-political characteristics of the territory, where control over resources by a group predominates, organizational fragility, and the invisibility of social actors such as the family and the community.^10^ All of this acts to the detriment of the capacities for taking advantage of opportunities present in the territory because, according to Zambrano and Henríquez,^16^ these conditions strengthen individualism and foster clientelistic relationships with local governments.

A second analytical axis is constituted by the individualistic vision of empowerment supported by those family narratives that gave rise to the categorical label: *“it’s the spark”,* to refer to the potential that families possess to undertake actions, self-confidence, and even faith in God to overcome diseases. This perspective emerged in the decades of the 1970s and 1980s of the 20th century, as an inherent part of the discourse on human well-being that prompted reading the link with self-esteem or personal fulfillment in response to human needs to interact or relate to each other.^39^

Regarding this, Cooke^40^ proposes interpreting the individualistic vision of empowerment as a concept that has undergone different interpretative forms ranging from self-expression, self-improvement, to entering the field of psychology in the form of self-determination of one’s life and a sense of personal control. This process is not encapsulated within the personal realm, as can be seen in the narratives of the interviewed families when they express the connection of this personal power with the need to manage matters of interest to the family, which, from Rappaport’s perspective,^14^ would translate into having the power to do something mediated by a condition of dominance based on that particular aspect.

Based on Rappaport’s reading,^14^ the notion of empowerment would be comprised of two essential elements: individual determination and democratic participation. The first element corresponds to the control that each person exercises over their own life, while the second integrates the democratic participation that occurs within a community, which generally seeks to defend legal rights, political power, among others, through the interconnection of participants with organizations such as schools, churches, and various social groups.

Sánchez’s reading^39^ enriches the dialogue of family narratives in Córdoba by establishing the existence of two forms of psychological empowerment: subjective and potential. The first constitutes an aspect of interest for achieving effective competence, as it represents an initial condition that allows individuals to develop collective actions to achieve objectives. This dimension is enhanced, in line with Zimmerman’s contributions^15^^)^ to the concept of empowerment, to the extent that individuals feel the need to liberate themselves from the effects of oppression, under the premise that individuality requires developing a sense of “self” to enhance individual capacity and self-confidence.

Up to this point, the family narratives constructed around empowerment by the interviewed families align with the interpretation of Ryynänen and Nivala,^41^ who point out two streams of thought for analyzing this category: the first, of a structuralist nature, related to the concept of power, and the second, situates it within the framework of the inner and individual strength of individuals, enabling them to act on a particular situation.

On the other hand, the narratives focused on “the example” as an educational mediation to achieve optimal levels of prevention and control of dengue fever. That is, by establishing channels of dialogue, arousing attention, and fostering preventive practices through modeling by parents and older adults. These aspects summarize the intention to act in the face of a problem present in the territory, which involves raising awareness, distributing tasks, and family leadership within and with the immediate environment, accompanied by the ability to persuade. Through persuasion, a motivated family with conceptual ownership of the topic would be capable of catalyzing empowerment processes, not only within their own group but also within other families in their community. These findings are similar to those of Garrido et al.,^42^ regarding the need for family groups to acquire knowledge to achieve higher levels of empowerment.

As Sánchez Vidal^39^^)^ reflects, the explicit incorporation of the power component introduced by the concept of empowerment has been primarily focused on the subjective empowerment dimension of individuals in micro-social spaces, postponing the influences of the broader socio-political context. While this can be a valuable contribution, it is necessary to be critical regarding the extent of the changes allowed by a globalized and dualized society. Therefore, in the field of prevention, we must address empowerment in this dual dimension. It seems particularly relevant, for example, to promote processes that allow questioning and transforming gender roles, as well as the need for organization and collective participation to advance deeper transformations that support a prevention system based on substantive changes in living conditions.

## CONCLUSIONS

In general terms, the narratives of empowerment constructed by family groups remain anchored to a metanarrative perpetuated by relationships of dominance/submission that restrict decision-making, the mobilization of knowledge, and family practices of disease prevention and control. These relationships are perceived at a micro level when, despite the prominent role of women in family health care, they resign themselves to male leadership due to economic provision; and at a community level, adherence to political or organizational power derived from the need to obtain economic benefits is observed.

Despite the above, regarding dengue prevention and control, narratives emphasizing the capacities of the family group to confront dengue stand out. This clearly demonstrates the domains for the development of an empowerment process at the family level, such as leadership, intra-family communication, understanding the dengue phenomenon, maintaining permanent alertness, dialoguing, and mobilizing the knowledge built in the community into their daily lives. These results contradict the metanarratives of the health sector that perpetuate the belief in the family image associated with passivity, submission, and disinterest, serving as a starting point to encourage reflection on new ways to address and treat the disease.

The challenge is also to construct mechanisms of family participation, communication, and governance to provide scenarios through which families can deploy their capacities in dengue prevention and control. This requires displacing metanarratives that guide public health policies focused on chemical vector control with little effectiveness in developing family educational processes to reverse the individualistic sense of intervention, replacing it with collective actions where families receive the necessary support on their path to empowerment.

In summary, the main limitation of the study stems from the nature of qualitative research itself, with an awareness that the extrapolation of our findings should be done cautiously when addressing other family groups in the territory whose sociodemographic characteristics do not align with the families that participated in this research. Nonetheless, it would be possible to consider, as a contribution to the understanding of the studied phenomenon, the values of association and governance present in rural family groups, as well as the extensive participation of older adults bearing knowledge and enriched experiences in disease management, which call for further studies in the field of family empowerment.
